# Long-term outcomes of catheter ablation for ventricular arrhythmias: comparing techniques with and without intracardiac echocardiography - what matters?

**DOI:** 10.1186/s12872-024-04056-x

**Published:** 2024-07-26

**Authors:** Mingjie Lin, Chuanzhen Ma, Bing Rong, Kai Zhang, Tongshuai Chen, Juntao Wang, Wenqiang Han, Jingquan Zhong, Lin Wu

**Affiliations:** 1 Department of Cardiology, Peking University First Hospital, Beijing, China; 2 State Key Laboratory for Innovation and Transformation of Luobing Theory, Key Laboratory of Cardiovascular Remodeling and Function Research, Department of Cardiology, Chinese Ministry of Education, Chinese National Health Commission and Chinese Academy of Medical Sciences, Qilu Hospital of Shandong University, No. 107 Wenhuaxi Road, Jinan, 250012 China; 3 Department of Cardiology, Cheeloo College of Medicine, Qilu Hospital (Qingdao), Shandong University, Qingdao, China; 4 Department of Geriatric Medicine, Cheeloo College of Medicine, Qilu Hospital, Shandong University, Jinan, China

**Keywords:** Premature ventricular complexes, Intracardiac echocardiography, Catheter ablation, Complication

## Abstract

**Background:**

The increasing use of intracardiac echocardiography (ICE) in the ablation of premature ventricular complexes (PVCs) has raised questions about its true efficacy and safety.

**Methods:**

This retrospective study collected the periprocedural complications and PVC burden post ablation. The risk factors of PVC recurrence was further explored.

**Results:**

The study included patients treated without ICE (control group, *n* = 451) and with ICE (ICE group, *n* = 155) from May 2019 to July 2022. The ICE group demonstrated significantly lower fluoroscopy times and X-ray doses. There were no major complications in the ICE group, and the difference in the occurrence of periprocedural complications between the groups was not statistically significant (*p* = 0.072). The long-term success rates were similar for the control and ICE groups (89.6% and 87.1%, respectively). The origin of PVCs was identified as the independent factor for ablation success.

**Conclusions:**

The use of ICE did not confer an advantage with regard to long-term success in PVCs ablation. To thoroughly evaluate the safety and effectiveness of ICE in PVCs ablation, a prospective, multicenter, randomized study is warranted.

**Supplementary Information:**

The online version contains supplementary material available at 10.1186/s12872-024-04056-x.

## Background

Premature ventricular complexes (PVCs) are frequently encountered cardiac arrhythmias that become more prevalent with advancing age. In fact, approximately 50% of individuals over 50 years of age, regardless of the presence of heart disease, exhibit PVCs during extended monitoring [1, 2]. Catheter ablation (CA) has proven to be more effective than pharmacological treatments in reducing PVC burden and enhancing both left and right ventricular function [3–5]. Approximately 80% of patients achieve long-term relief following CA, although the procedure entails a 4% risk of complications. Outcomes are typically less favorable for PVCs originating from non-outflow tract (non-OT) locations, and there is a significant need to improve the success rates of CA for these cases [3, 4, 6]. Intracardiac echocardiography (ICE) provides high-quality, real-time imaging of the heart and has been extensively adopted in both diagnostic and therapeutic cardiac interventions [7]. Observational data have indicated that the application of ICE during ventricular tachycardia (VT) ablation procedures correlates with a reduced incidence of major complications [8, 9]. According to the latest expert consensus on ICE, its use could be particularly beneficial for the catheter ablation of non-OT origin PVCs [7]. Nonetheless, it remains to be determined whether ICE-assisted CA leads to better outcomes or a decrease in complications for patients with frequent PVCs. The goal of the current study is to evaluate the immediate and long-term success as well as the periprocedural complication rates of CA in patients with frequent PVCs or non-sustained VT, comparing outcomes between those who underwent ICE-assisted CA and those who did not.

## Methods

This retrospective study received approval from the local institutional Ethics Committee, and all participants provided written informed consent. We included all patients who underwent CA for PVCs or non-sustained VT consecutively from May 2019 to July 2022, barring those who met any of the exclusion criteria, which consisted of: (i) an insignificant number of PVCs or non-inducible VT during the procedure, or (ii) follow-up duration of less than 3 months or lost to follow-up. The patients were segregated into two cohorts: the control group who did not utilize ICE, and the ICE group wherein ICE was employed.

### Follow-up strategy

Following discharge, it was recommended that patients undergo at least one cardiac rhythm evaluation through electrocardiography (ECG) or 24-hour Holter monitoring at the 1-month mark post-procedure, and a transthoracic echocardiography 3 months afterwards to check for abnormalities in the left ventricular end-diastolic diameter (LVEDD) or left ventricular ejection fraction (LVEF).

For the purpose of this study, we contacted patients to gather current symptomatology, the outcomes from ECG and 24-hour Holter monitoring, the most recent measurements of LVEDD and LVEF, post-CA complications, incidences of rehospitalization, and details regarding pharmacotherapy. Baseline data, including patient demographics, PVC burden, time elapsed since initial diagnosis, history of pharmacotherapy, and cardiovascular history, were extracted from the electronic medical records system. Antiarrhythmic drugs were categorized according to the updated classification system [10], and traditional Chinese medicine treatments such as Shensong Yangxin [11] capsules and Wenxin Keli [12] were also accounted for.

### Catheter ablation procedure

Antiarrhythmic medications were discontinued at least three days prior to the procedure, which was carried out by seasoned electrophysiologists. Fluoroscopy, set at 7–15 frames per second, facilitated visualization. In cases where PVCs were scarce or absent at baseline, the procedure proceeded only if PVCs emerged frequently following intravenous administration of isoproterenol. For non-sustained VT, either programmed ventricular stimulation or isoproterenol infusion would be used to induce VT. The decision to employ ICE with a frequency range of 5–10 MHz (Johnson and Johnson, Inc.) was made during pre-procedure consultations between patients and their doctors.

Ablation targeted the site of earliest ectopic activation, utilizing an open-irrigation catheter with a tip size of either 3.5–4.0 mm (Celsius, Johnson and Johnson, Inc.; Coolffex, St. Jude Medical Inc.) at settings typically ranging from 30 to 45 W and a maximum temperature of 45 °C. In conjunction with a 3D electroanatomic mapping system (CARTO 3, Johnson and Johnson, Inc.), ideal ablation sites were identified based on local activation time, a unipolar QS-pattern, and the presence of reversed polarity [13]. PVC origin was classified as originating from a variety of anatomical locations such as the right ventricular outflow tract (RVOT), left ventricular outflow tract (LVOT), papillary muscles, tricuspid or mitral annulus, epicardium or LV summit, and areas adjacent to the His bundle and left bundle branch.

ICE was conducted as per established guidelines [7]. Through bilateral femoral vein access, images of the annulus, right ventricle, aortic long axis, RVOT, and LVOT long axis were obtained in the right atrium view; whereas images of the right ventricle, LV, moderator band, interventricular septum, and papillary muscles were captured in the right ventricular view (refer to Supplementary Fig. 1). Radiofrequency energy levels (with a 3.5 mm-tip open-irrigation deflectable catheter manufactured by Johnson and Johnson Inc. or Coolflex by St. Jude Medical Inc.;)—ranging from 30 to 45 W, depending on the ablation site (with a maximum temperature of 45 °C, a flow rate of 20–30 mL/min, and a duration of 90 to 180 s)—were used for ablation. A site was designated as the origin if PVCs ceased within 5 s of energy application.

Acute procedural success was defined as the abolition of the targeted PVCs for a minimum of 30 min post-procedure, inclusive of isoproterenol infusion and programmed electrical stimulation [4, 13]. The success of the treatment was evaluated based upon 80% reduction in PVC burden, a commonly adopted criterion in literature [4]. Complications were categorized as either major or minor, the former requiring procedural intervention, blood transfusion, prolonged hospitalization, or resulting in enduring clinical detriment.

### Statistical analysis

Data analysis was conducted using SPSS version 22.0 (IBM Corp.) and Prism version 8.0.2 (Graphpad Software, Inc.), adopting a significance threshold of *P* < 0.05. The differences in cumulative survival without PVCs or VT between the control group and the ICE group are depicted using Kaplan-Meier curves. To ascertain predictors of both immediate and sustained success, logistic regression analyses were employed. The findings are presented as odds ratios (ORs) with corresponding 95% confidence intervals (CIs). A comprehensive list of potential confounders was considered, including age, sex, ICE utilization, origins of PVCs, PVC burden, duration of PVCs, abnormal LVEF, abnormal LVEDD, left atrial diameter of 40 mm or greater, cardiovascular comorbidities, smoking habits, alcohol intake, and obesity (defined as a body mass index of 28.0 or higher). Variables that reached a significance level of *P* < 0.10 in the initial analyses were incorporated into a multivariable logistic regression model to identify independent predictors.

## Results

**Fig. 1:**
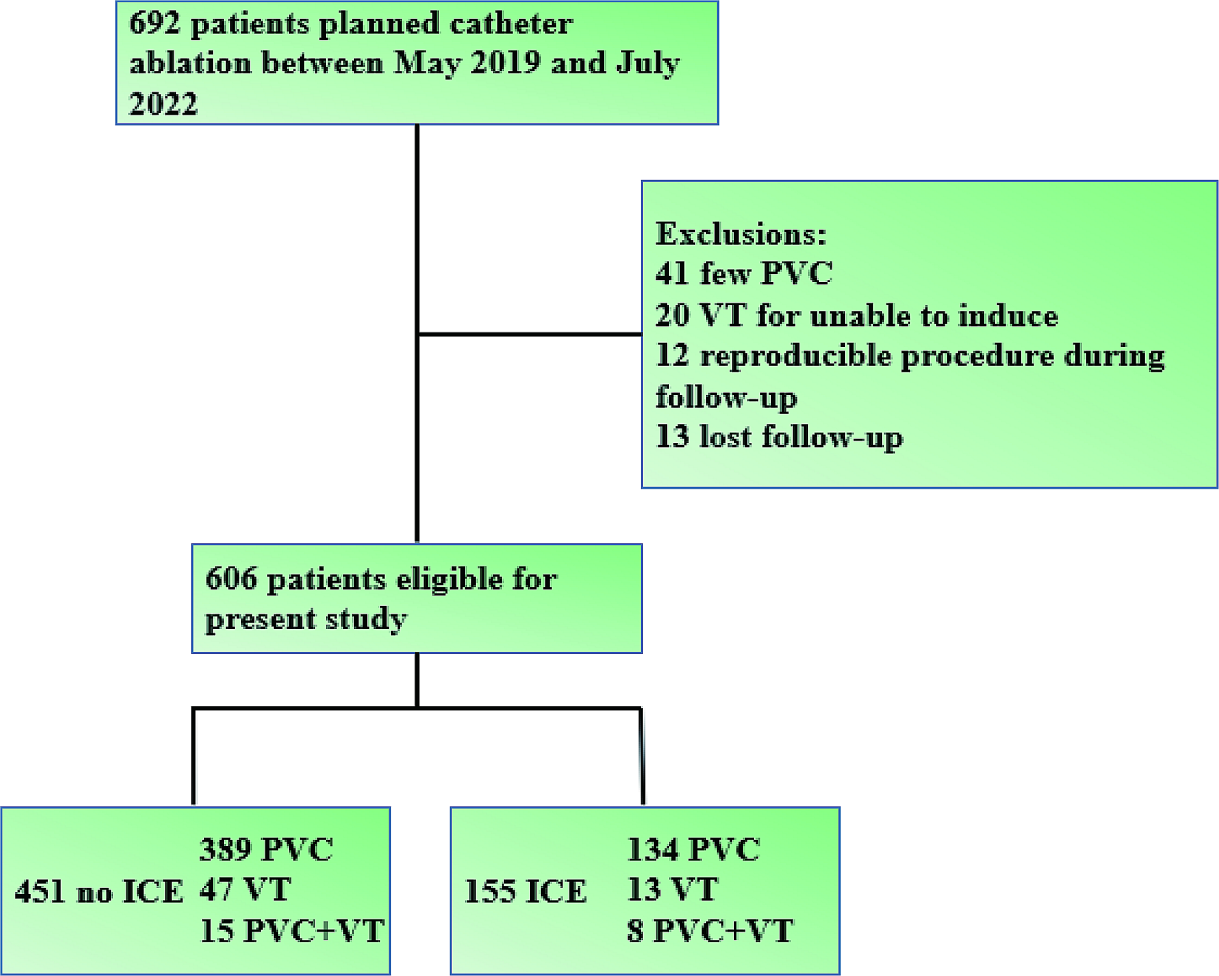
Flowchart of study patients with ventricular arrhythmias. PVC: premature ventricular complexes; VT: ventricular tachycardia; ICE: intracardiac echocardiography

Figure 1 illustrates that out of the 692 patients who intended to undergo CA from May 2019 to July 2022, 606 patients qualified for inclusion in the present analysis: 451 were in the control group and 155 were in the ICE group. As detailed in Table 1, there were no significant differences in baseline characteristics between the two groups, except for sex distribution and potassium levels. Approximately 60% of patients had a PVC burden of 20% or more; a majority of patients (89.6%) experienced symptoms related to PVCs or VT; 11.1% of patients exhibited a LVEF of less than 55%, and 15.5% had an abnormal LVEDD.

**Table 1:** Baseline and follow-up information

	Control (*n* = 451)	ICE (*n* = 155)	P value	
Baseline data				
Age, mean (SD), years	46.2 (16.1)	46.7 (15.2)	0.195	
Male, n(%)	179 (39.7)	76 (49.0)*	0.048	
Type of arrhythmia			0.471	
PVC, n(%)	389 (86.3)	134 (86.5)		
VT, n(%)	47 (10.4)	13 (8.4)		
PVC and VT, n(%)	15 (3.3)	8 (5.2)		
Burden of PVC			0.925	
<20%, n(%)	163 (40.3)	55 (38.7)		
20–30%, n(%)	136 (33.7)	48 (33.8)		
>30%, n(%)	105 (26.0)	39 (27.5)		
Symptoms				
Palpitation, n(%)	344 (76.3)	118 (76.1)	1.000	
Chest distress, n(%)	255 (56.5)	80 (51.6)	0.123	
Chess pain, n(%)	93 (20.6)	35 (22.6)	0.648	
Duration since dignosis (SD), months	37.7 (56.9)	38.3 (58.4)	0.908	
Coronary heart disease, n(%)	75 (16.6)	25 (16.1)	1.000	
Hypertension, n(%)	117 (25.9)	44 (28.4)	0.598	
Diabetes mellitus, n(%)	37 (8.2)	15 (3.3)	0.618	
Prior stroke/TIA/systemic embolism, n(%)	25 (5.5)	6 (3.9)	0.528	
Abnormal left ventricular ejection fraction, n(%)	43(9.5)	21(13.5)	0.173	
Abnormal left ventricular diastolic diameter, n(%)	58(12.8)	27 (17.4)	0.180	
Smoking, n(%)	76 (16.9)	30 (19.4)	0.465	
Alcohol consumption, n(%)	67 (14.9)	38 (24.5)*	0.009	
Body mass index ≥ 28.0, n(%)	87 (19.3)	35 (22.6)	0.416	
Abnormal troponin I, n(%)	58 (12.9)	25 (16.1)	0.343	
Abnormal potassium, n(%)	25 (5.5)	1 (0.6)*	0.006	
Antiarrhythmic drugs				
Class Ic, n(%)	8 (1.8)	7 (4.6)	0.072	
Class IIa, n(%)	131 (29.0)	51 (33.8)	0.363	
Class IIIa, n(%)	10 (2.2)	3 (1.9)	1.000	
Others, n(%)	54 (12.0)	22 (14.6)	0.483	
Follow-up data				
Follow-up duration, mean (SD), months	24.4 (10.4)	11.3 (6.6)*	0.000	
24 h-holter monitoring	390 (86.5)	136 (87.7)	0.784	
Improvement of left venricular ejction fraction, mean (SD), %	7.26 (7.07)	5.62 (5.50)	0.337	
Reduction of left ventricular diastolic diameter, mean (SD), mm	3.38 (3.69)	2.67 (2.83)	0.377	
Cardiovascular rehospitalization, n(%)	26 (5.8)	3 (1.9)	0.078	
Antiarrhythmic drugs*				
Class Ic, n(%)	9 (2.0)	8 (5.2)*	0.049	
Class IIa, n(%)	51 (11.3)	26 (16.8)	0.093	
Class IIIa, n(%)	10 (2.2)	3 (1.9)	1.000	
Others, n(%)	20 (4.4)	17 (11.3)*	0.012	

Abbreviations: ICE: intracardiac echocardiography; PVC: premature ventricular complexes; VT: ventricular tachycardia.

* *P* < 0.05, compared with control group

**Fig. 2:**
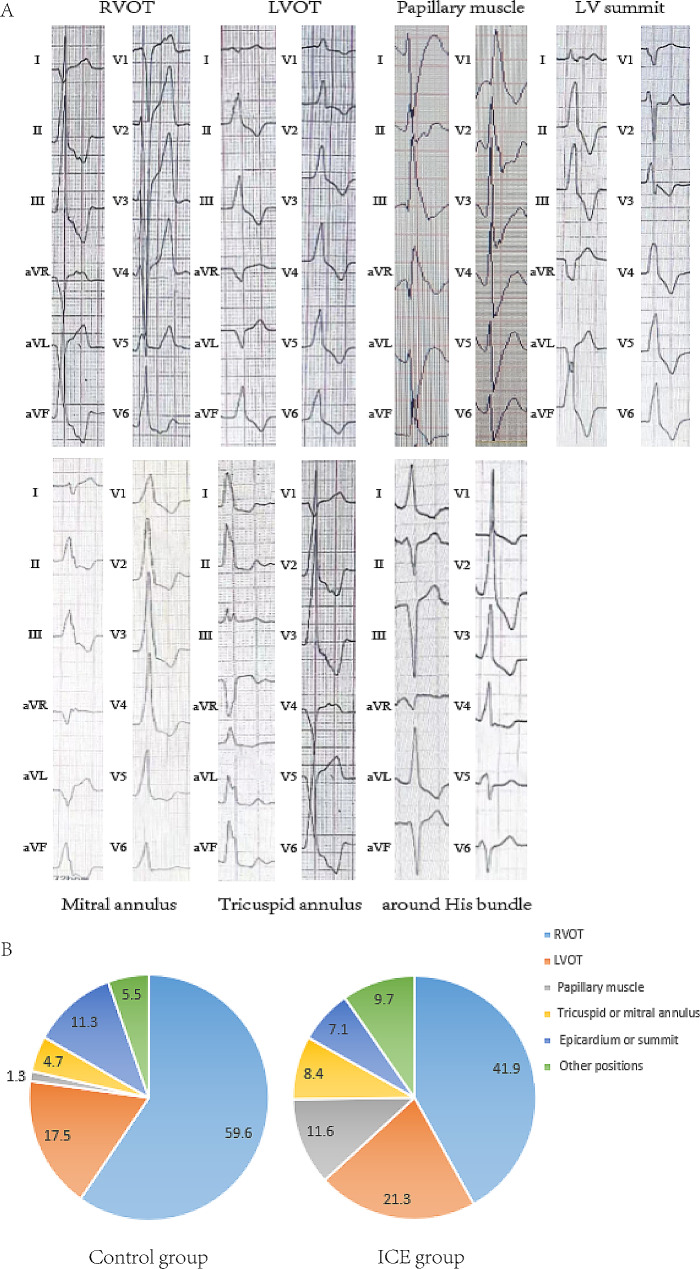
The representative 12-lead electrocardiograms of ventricular arrhythmias from different origins (A) and distribution of origins (B). RVOT: right ventricular outflow tract; LVOT: left ventricular outflow tract

In comparing the control group, it was observed that the origin sites of the condition were more frequently detected outside RVOT in the ICE group, as shown in Fig. 2. The ICE group experienced a longer procedural duration; however, they benefited from reduced fluoroscopy times and lower X-ray exposure. The acute success rates between the two groups were similar. In the control group, four significant complications were documented: two instances of transient ischemic attacks, one case of ischemic stroke, and one episode of ventricular fibrillation that transpired during the procedure. Notably, there were no reports of cardiac tamponade or any other severe complications in either group. While the rates of complications varied, the differences did not reach statistical significance, with a p-value of 0.072, as presented in Table 2.

**Table 2:** Procedural outcomes

	Control (*n* = 451)	ICE (*n* = 155)	P valure	
Origins			0.000	
RVOT, n(%)	269 (59.6)	65 (41.9)*	0.000	
LVOT, n(%)	79 (17.5)	33 (21.3)	0.337	
Papillary muscle, n(%)	6 (1.3)	18 (11.6)*	0.000	
Tricuspid or mitral annulus, n(%)	21 (4.7)	13 (8.4)	0.104	
Epicardium or summit, n(%)	55 (11.3)	11 (7.1)	0.099	
Other positions, n(%)	25 (5.5)	15 (9.7)	0.090	
Total procedure time, mean (SD), minutes	103.9 (47.2)	115.6 (51.0)*	0.009	
Total ablation time, mean (SD), seconds	170.5 (94.1)	173.5 (89.7)	0.727	
Fluoroscopy time, mean (SD), seconds	129.1 (85.8)	83.8 (70.4)*	0.000	
Dose of X-ray, mean (SD), mGy	12.15 (8.2)	7.9 (6.7)*	0.000	
Number of ablation lesions, n(%)	1.66 (0.94)	1.52 (0.82)	0.099	
Instant results			0.548	
Success, n(%)	377 (83.4)	130 (83.4)		
Partial response, n(%)	35 (7.8)	15 (9.7)		
Failure, n(%)	39 (8.6)	10 (6.5)		
Complications			0.072	
Major, n(%)	4 (0.9)	0 (0)		
Minor, n(%)	72 (16.0)	15 (9.7)		
Length of stay post procedure, mean (SD), days	1.99 (2.35)	1.56 (0.95)*	0.027	

Abbreviations: ICE: intracardiac echocardiography; RVOT: right ventricular outflow tract; LVOT: left ventricular outflow tract.

* *P* < 0.05, compared with control group

**Fig. 3:**
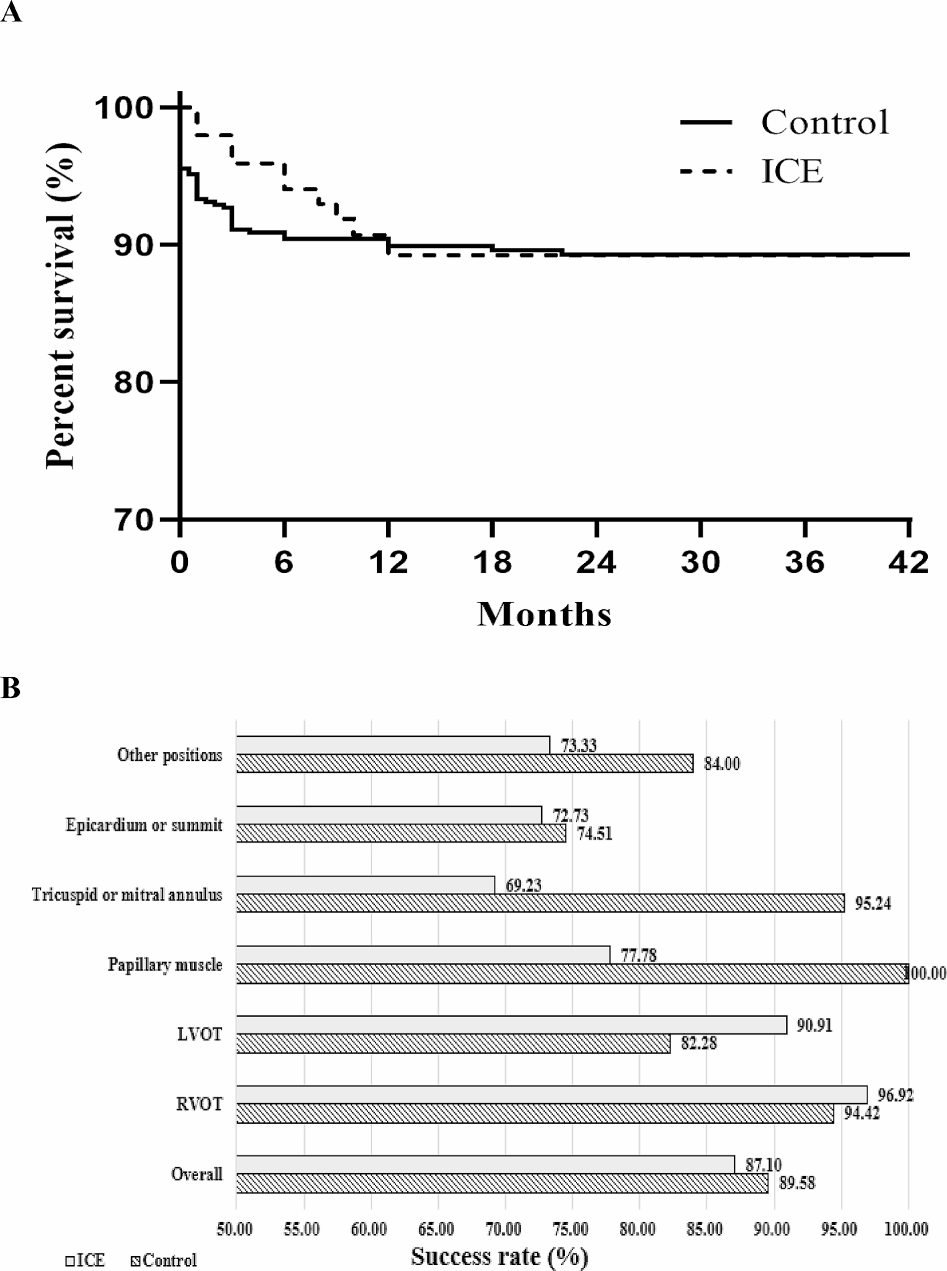
Kaplan-Meier curves for cumulative survival free from long-term outcome between control and ICE group (A) and long-term success rate from different origins between control and ICE groups (B). ICE: intracardiac echocardiography; RVOT: right ventricular outflow tract; LVOT: left ventricular outflow tract

The follow-up duration was considerably longer for the control group (Table 1). More than 85% of the patients underwent at least one 24-hour Holter monitoring session, and the remaining patients all underwent at least one ECG examination. (Table 1). The use of antiarrhythmic drugs was less frequent. CA led to a significant increase in LVEF and a decrease in LVEDD during the follow-up period. Notably, the LVEF improvement was more pronounced in the control group. The rate of cardiovascular rehospitalization was similar between the groups throughout the follow-up, and there were no reported deaths (as detailed in Table 1). As depicted in Fig. 3A, the long-term success rates were comparable between the control and the ICE groups, with success rates of 89.6% and 87.1%, respectively. Success rates for RVOT origins exceeded 90% in both groups. As shown in Fig. 3B, there was no significant difference in the long-term success rates from various ablation sites between the groups.

**Fig. 4:**
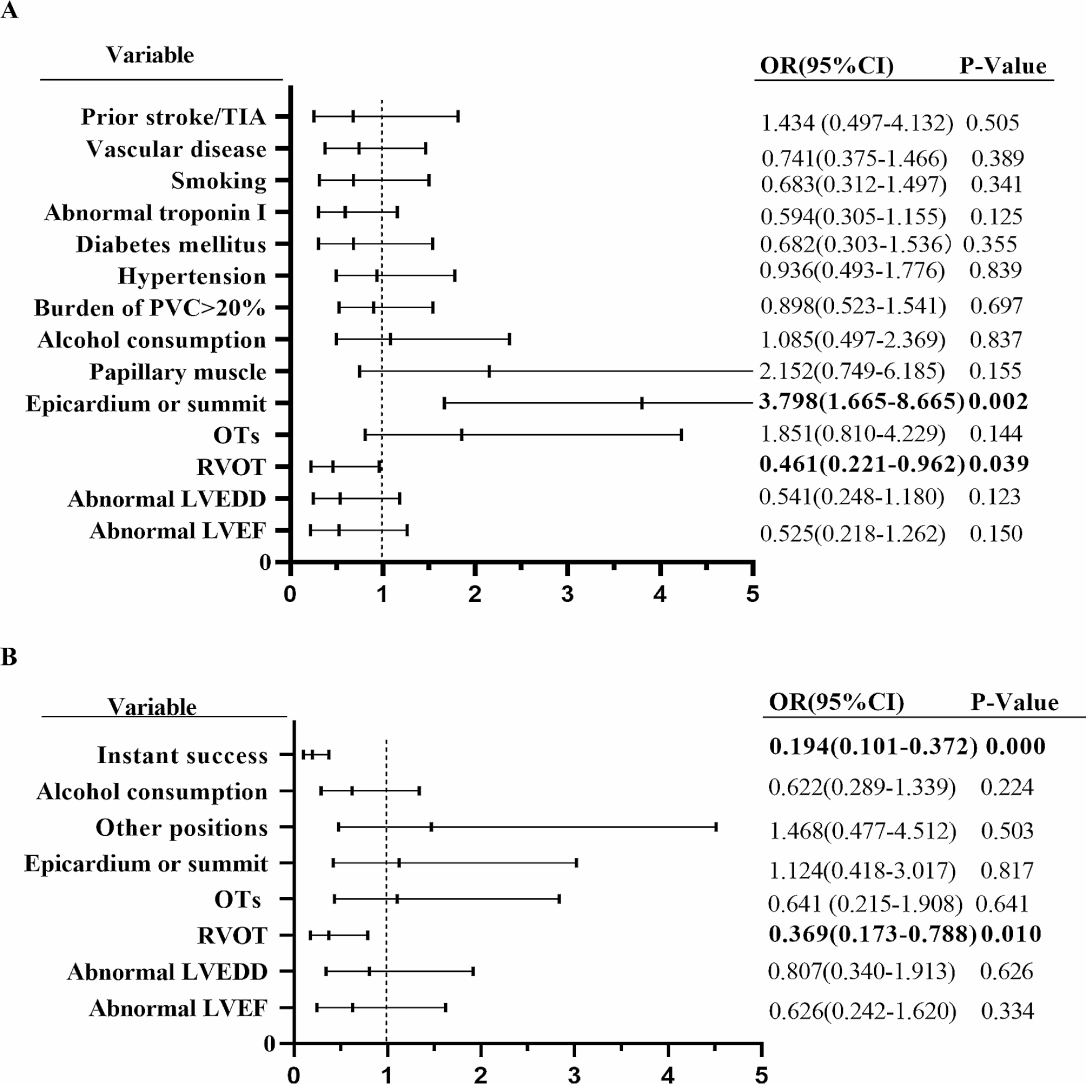
Multivariate analysis for predictors of acute procedural success (A) and long-term success (B). LVEF: left ventricular ejection fraction; LVEDD: left ventricular end-diastolic diameter; PVC: premature ventricular complexes; RVOT: right ventricular outflow tract; LVOT: left ventricular outflow tract; OTs: outflow tracts; TIA: transient ischemic attack

Table 3 presents the univariable logistic regression analysis, which identified cardiovascular comorbidities, abnormal LV function and size, papillary muscle involvement, and ablation origins from the epicardium or summit as factors associated with lower acute success rates. Conversely, being female and having an RVOT origin of the arrhythmia were associated with higher acute success rates. The multivariable logistic regression analysis, adjusted for age and gender, revealed that RVOT and epicardium or summit origins were independent predictors of acute success (as shown in Fig. 4A). Regarding long-term outcomes (Table 4), having an RVOT origin and achieving immediate success were significant predictors of better outcomes, as illustrated in Fig. 4B.

**Table 3:** Univariable and multivariable logistic regression for acute success of catheter ablation

Variable	Univariate		Multivariate		
	OR (95%CI)	*P*-value	OR (95%CI)	*P*-value	
Age	1.039 (1.023–1.055)	0.000	1.016(0.995–1.037)	0.138	
Female sex	0.547 (0.355–0.845)	0.006	0.904(0.468–1.745)	0.763	
ICE use	0.979 (0.597–1.608)	0.935			
Abnormal LVEF	4.651 (2.660–8.197)	0.000	0.525(0.218–1.262)	0.150	
Abnormal LVEDD	3.891 (2.375–6.410)	0.000	0.541(0.248–1.180)	0.123	
Burden of PVC > 20%	1.572 (1.008–2.457)	0.046	0.898(0.523–1.541)	0.697	
Origins					
RVOT	0.145 (0.085–0.246)	0.000	0.461(0.221–0.962)	0.039	
LVOT	0.976 (0.559–1.705)	0.933			
OTs	0.143 (0.090–0.227)	0.000	1.851(0.810–4.229)	0.144	
Papillary muscle	4.750 (2.062–10.941)	0.000	2.152(0.749–6.185)	0.155	
Tricuspid or mitral annulus	1.104 (0.445–2.740)	0.832			
Epicardium or summit	10.571 (5.986–18.669)	0.000	3.798(1.665–8.665)	0.002	
Other positions	1.535 (0.707–3.335)	0.278			
Obesity	1.429 (0.862–2.364)	0.166			
Duration since dignosis	1.000 (0.996–1.004)	0.995			
Hypertension	2.532 (1.618–3.968)	0.000	0.936(0.493–1.776)	0.839	
Diabetes mellitus	2.525 (1.339–4.762)	0.004	0.682(0.303–1.536)	0.355	
Prior stroke/TIA /systemic embolism	3.040 (1.408–6.579)	0.005	0.678(0.254–1.811)	0.438	
Vascular disease	3.086 (1.883–5.051)	0.000	0.741(0.375–1.466)	0.389	
Smoking	2.463 (1.506–4.032)	0.000	0.683(0.312–1.497)	0.341	
Alcohol consumption	1.684 (1.003–2.825)	0.049	1.085(0.497–2.369)	0.837	
Abnormal troponin I	1.898 (1.095–3.289)	0.022	0.594(0.305–1.155)	0.125	
Abnormal potassium	1.658 (0.645–4.255)	0.295			

Abbreviations: ICE: intracardiac echocardiography; LVEF: left ventricular ejection fraction; LVEDD: left ventricular end-diastolic diameter; PVC: premature ventricular complexes; RVOT: right ventricular outflow tract; LVOT: left ventricular outflow tract; OTs: outflow tracts; TIA: transient ischemic attack.

**Table 4:** Univariable and multivariable logistic regression for long-term outcome of catheter ablation

Variable	Univariate		Multivariate	
	OR (95%CI)	*P*-value	OR (95%CI)	*P*-value
Age	0.999 (0.983–1.015)	0.897	0.983(0.963–0.998)	0.026
Female sex	1.483 (0.891–2.466)	0.129	0.787(0.410–1.511)	0.472
ICE use	0.785 (0.449–1.373)	0.396		
Abnormal LVEF	3.367 (1.776–6.369)	0.000	0.626(0.242–1.620)	0.334
Abnormal LVEDD	2.674 (1.499–4.762)	0.000	0.807(0.340–1.913)	0.626
Burden of PVC > 20%	1.109 (0.664–1.848)	0.694		
Origins				
RVOT	0.238 (0.134–0.424)	0.000	0.369(0.173–0.788)	0.010
LVOT	1.589 (0.878–2.876)	0.126		
OTs	0.318 (0.189–0.533)	0.000	1.103(0.430–2.833)	0.838
Papillary muscle	1.648 (0.546–4.974)	0.376		
Tricuspid or mitral annulus	1.418 (0.530–3.798)	0.487		
Epicardium or summit	3.362 (1.777–6.362)	0.000	1.124(0.418–3.017)	0.817
Other positions	2.148 (0.946–4.879)	0.068	1.468(0.477–4.512)	0.503
Obesity	1.404 (0.779–2.538)	0.712		
Duration since dignosis	0.998 (0.994–1.003)	0.517		
Hypertension	1.200 (0.688–2.096)	0.519		
Diabetes mellitus	0.469 (0.142–1.548)	0.214		
Prior stroke/TIA /systemic embolism	0.855 (0.253–2.890)	0.802		
Vascular disease	1.119 (0.575–2.174)	0.742		
Smoking	1.420 (0.766–2.632)	0.265		
Alcohol consumption	1.742 (0.961–3.165)	0.068	0.622(0.289–1.339)	0.224
Abnormal troponin I	0.826 (0.380–1.799)	0.630		
Abnormal potassium	1.101 (0.321–3.788)	0.878		
Instant success	0.291 (0.209–0.405)	0.000	0.194(0.101–0.372)	0.000

Abbreviations: ICE: intracardiac echocardiography; LVEF: left ventricular ejection fraction; LVEDD: left ventricular end-diastolic diameter; PVC: premature ventricular complexes; RVOT: right ventricular outflow tract; LVOT: left ventricular outflow tract; OTs: outflow tracts; TIA: transient ischemic attack

## Discussion

ICE with three-dimensional electroanatomical mapping is increasingly utilized in the management of ventricular arrhythmias [14]. Although there is a paucity of evidence regarding its safety and efficacy for PVCs, our study sought to examine the associated complications and both the acute and long-term success of PVC CA with and without ICE. Our findings reveal that (i) CA was effective in eliminating PVCs or non-sustained VT, boasting an approximate 90% long-term success rate; (ii) the usage of ICE did not enhance acute or long-term outcomes; and (iii) ICE may be associated with a lower incidence of perioperative complications, albeit not reaching statistical significance.

Idiopathic ventricular arrhythmias, such as PVCs and VT, commonly originate from OT, particularly RVOT. As substantiated by previous reports and corroborated by our data, CA for PVCs stemming from the RVOT had the highest success rate at around 90% and a low rate of complications [4, 6]. For non-OT PVC origins, success rates were between 50 and 80%, with poorer outcomes observed from epicardial sites, aligning with our findings [4, 6]. PVC-induced cardiac dysfunction is considered a significant prognostic event for idiopathic ventricular arrhythmias [15]. In our study, 17.6% of patients demonstrated abnormal LVEF or LVEDD. Successful PVC elimination by CA was found to improve LVEF and decrease LVEDD, consistent with results from other studies [3–5, 16].

ICE provides high-resolution, real-time visualization of intracardiac structures and catheters during interventional procedures, assisting operators in monitoring lesion formation and characteristics [7]. The benefits of ICE include enhanced patient tolerance, as well as reduced radiation and contrast agent exposure, echoing the ALARA (as low as reasonably achievable) principle [7]. ICE-guided procedures for conditions such as atrial fibrillation and left atrial appendage closure are increasingly being used due to their safety and efficacy [17, 18]. Although comparative studies are scarce, ICE is believed to heighten the safety and effectiveness of PVC CA in comparison to traditional mapping technologies.

Michael and colleagues reported that in patients with a history of implantable cardioverter defibrillator or cardiac resynchronization therapy who underwent VT ablation, the usage of ICE was associated with a decreased 12-month risk of VT-related readmission and a reduced need for repeat VT ablation. Interestingly, this intervention did not significantly alter complication rates [8]. A Japanese nationwide observational study also found that ICE application notably reduced the risk of cardiac tamponade, although it did not present additional clinical benefits for other safety outcomes or effectiveness [9].

Clinically, long-term success rates are of immense concern. Factors affecting outcomes primarily include PVC origin and burden [4, 6], with RVOT-derived arrhythmias generally showing better prognosis and epicardial origins being associated with higher recurrence [4]. In our study, PVC origin was a principal determinant of ablation outcomes as well.

ICE use has been increasingly utilized in PVCs ablation. CA of PVCs originating from LV summit is challenging. However, utilizing ICE, Santiago et al. achieved an 84% acute success rate with no complications in a cohort of 26 patients receiving non-fluoroscopic CA [19]. Traditional three-dimensional (3D) electroanatomical mapping was less effective for papillary PVCs ablation. Lin and colleagues showed that augmenting this technique with ICE and ICE-generated 3D cardiac anatomy can raise the acute success rate above 90% [20]. While in present study, ICE use does not affect success rates, which may be related to the fact that all the patients we selected have idiopathic PVCs. The ICE group may have involved more complex origin sites, and non-RVOT origins were associated with poorer outcomes [4]. Despite the challenges in ablating originating from specific locations in the heart such as the papillary muscles and the left ventricle summit [21], we have found in clinical practice that, for experienced operators, the difficulty of catheter stability and positioning is related to the site of origin. The success rate of ablation often depends on the depth of the lesion, and the use of ICE cannot solve this problem. Consequently, ICE utilization for idiopathic ventricular arrhythmias may offer limited assistance in substrate identification.

Despite the absence of comparative studies, there is empirical evidence suggesting that ICE usage may enhance the efficacy and safety of PVCs CA and minimize radiation exposure compared to traditional 3D electroanatomical mapping. In our study, we found that ICE application not only reduced the X-ray dose [20] but also, while the acute success rate and long-term outcomes did not significantly improve, the incidence of periprocedural complications did not significantly differ from the control group. Furthermore, the ICE group had a longer procedure duration. Although not statistically significant, the ICE group showed a trend towards fewer ablation lesions and a shorter average postoperative hospital stay, potentially enhancing efficiency and compensating somewhat for the cost of ICE equipment.

PVCs are frequently encountered in clinical practice, and CA is an effective method to alleviate symptoms and prevent cardiomyopathy associated with PVCs. ICE is becoming increasingly prevalent due to its proven benefits in managing atrial fibrillation and VT in patients with structural heart disease. Our findings underscore that in the ablation of idiopathic PVCs, while ICE did not significantly enhance success rates or reduce complications, the efficacy of the ablation procedure often depended on the precise location and depth of the PVCs. The study also observed longer procedure times with less favorable outcomes, emphasizing the importance of identifying the optimal ablation target swiftly to minimize procedural delays.

Our study is subject to certain limitations. Firstly, as a retrospective observational study, there is a potential for operator bias, particularly as the use of intracardiac echocardiography (ICE) may be preferentially chosen for complex lesions. This could skew the results. Additionally, the follow-up process for Holter monitoring and echocardiography was not standardized, raising the possibility of follow-up attrition and the potential to misclassify endpoint events. Despite this, the immediate success rate appears to have the most significant influence on recurrence, suggesting that variations in Holter follow-up may exert minimal impact on the long-term outcomes. Secondly, the relatively small sample size limited our ability to perform propensity score matching for variables between the study groups, which might have influenced the observed incidence of endpoint events. Thirdly, our analysis did not incorporate the morphology and duration of QRS complexes as potential factors for assessing the risk associated with procedural outcomes. These ECG characteristics are indicative of the origins and depth of PVCs and have been linked to ablation success in univariate analyses. Hence, the baseline QRS characteristics could potentially serve as preliminary indicators of ablation difficulty in clinical practice. In consideration of these issues, future prospective randomized controlled trials are warranted to clarify the definitive impact of ICE on the success and complications related to PVC ablation.

Overall, while ICE assists in minimizing fluoroscopic doses during PVC or non-sustained VT ablation, it does not appear to significantly enhance acute and long-term success rates or substantially decrease complication rates.

### Electronic supplementary material

Below is the link to the electronic supplementary material.

Supplementary Material 1

## Data Availability

The data underlying this article will be shared on reasonable request to the corresponding author.

## Declarations

### Clinical trial number

This is a retrospective study, and since it did not impact the treatment methods, it was not registered as a clinical trial.
