# Supplementary material for: Long-term outcomes of catheter ablation for ventricular arrhythmias: comparing techniques with and without intracardiac echocardiography - what matters?
Source: BMC Cardiovasc Disord. 2024 Jul 26;24:386. doi: 10.1186/s12872-024-04056-x (PMC11282682; doi:10.1186/s12872-024-04056-x)
Supplement: Supplementary file 1 — Supplementary Material 1 [file 12872_2024_4056_MOESM1_ESM.docx]

Supplementary Figure 1


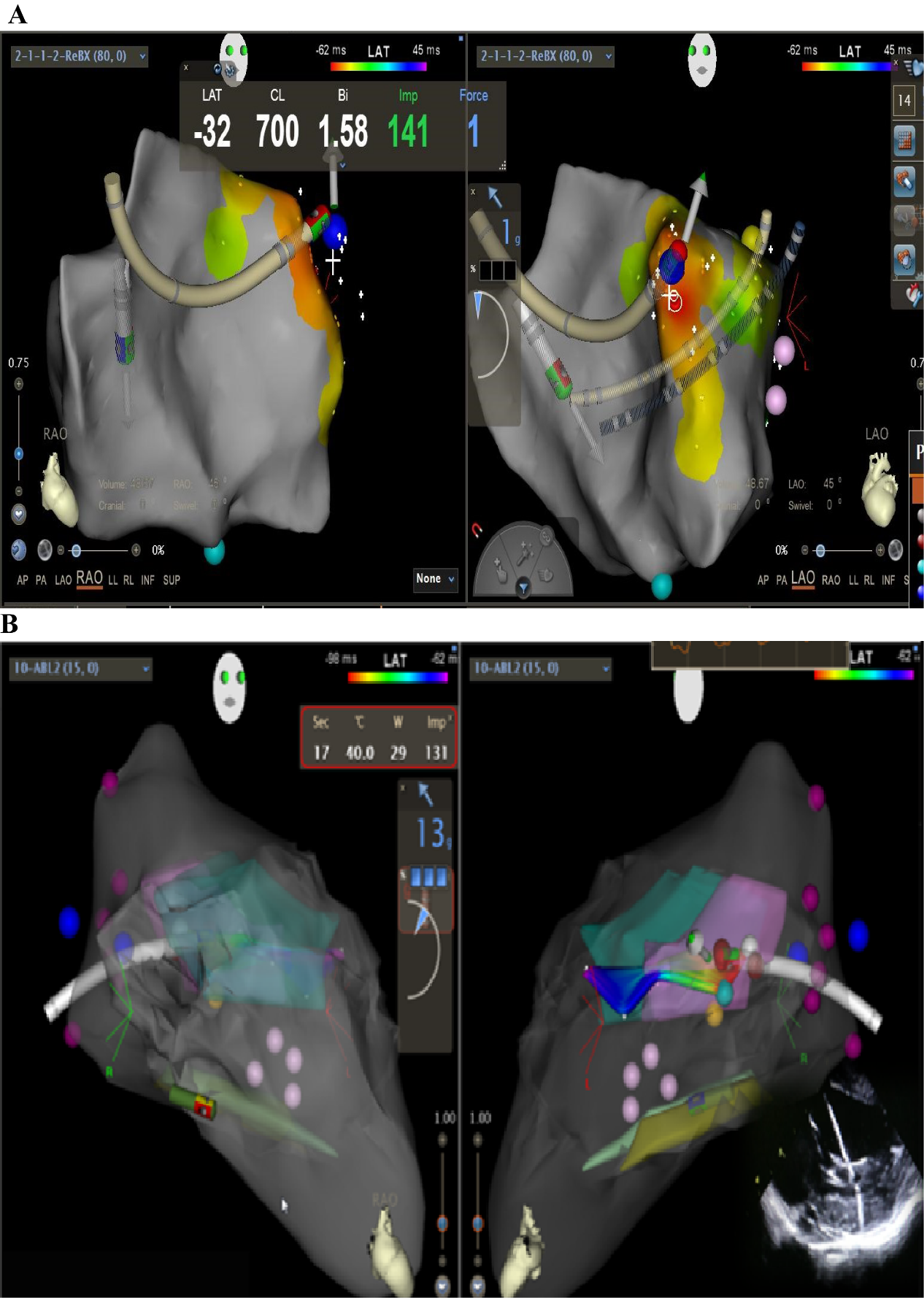


**Supplementary Figure 1**. Ventricular arrythmias ablation from left anterolateral papillary muscle with 3D electroanatomical map (CARTO 3, Johnson and Johnson, Inc.) only or in combination with intracardiac echocardiography use (5–10 MHz, Johnson and Johnson, Inc.) was decided by consultation between patients and doctors before procedure. A: intracardial structure was constructed with an adjustable catheter. At the blue points, the local activation time was the earlier than the referenced point about 62ms and than the earliest activation point of surface ECG about 32ms. Ventricular tachycardia eliminated within 3 seconds after ablation and without recurrence during 30 minutes observation time. B: intracardial structure was constructed with intracardiac echocardiography and ablation catheter. As presented in the lower right corner, papillary muscle and catheter were real-time observation. Ventricular premature complexes were eliminated after identifying the ideal site.
